# Molecular genetic investigation of hereditary breast and ovarian cancer patients in the Southern Transdanubian region: widening the mutation spectrum and searching for new pathogenic variants using next-generation methods

**DOI:** 10.3389/pore.2024.1611813

**Published:** 2024-08-01

**Authors:** László Baráti, Anita Maász, Alexandra Mikó, Éva Bércesi, Sultan Al Kalbani, Judit Bene, Sebestyén Kovács, László Mangel, Kinga Hadzsiev

**Affiliations:** ^1^ Department of Medical Genetics, Clinical Centre, Medical School, University of Pécs, Pécs, Hungary; ^2^ Institute for Translational Medicine, Medical School, University of Pécs, Pécs, Hungary; ^3^ Department of Oncotherapy, Clinical Centre, Medical School, University of Pécs, Pécs, Hungary; ^4^ Urology Clinic, Clinical Centre, University of Pécs, Pécs, Hungary

**Keywords:** HBOC, new generation sequencing, multigene panel testing, BRCA1, BRCA2

## Abstract

Hereditary breast and ovarian cancer is a well-known genetic condition, inherited mainly in an autosomal dominant way, which elevates the risk of developing malignancies at a young age in heterozygous carriers. Advances in new generation sequencing have enabled medical professionals to determine whether a patient is harbouring mutations in moderate- or high penetrance susceptibility genes. We conducted a retrospective analysis among 275 patients who underwent genetic counselling and multigene panel testing for hereditary breast and ovarian cancer syndrome in our department. From these patients 74.5% (205/275) were affected by some type of malignancy, while the remaining 25.5% (70/275) had a positive family history of different cancers, suggesting a genetic predisposition. These tests confirmed a genetic variant in 29.8% and 28.6% of these patient groups respectively. The results also mirrored our general knowledge concerning the genetic background of hereditary breast and ovarian cancer, as variants in either one of the *BRCA1* and *BRCA2* genes proved to be the most common cause among our patients with 41.5%. Our test also detected a novel mutation in the *CDH1* gene and three patients with double heterozygosity in two different susceptibility genes. This study demonstrates the relevance of genetic counselling and non-*BRCA* gene sequencing among cancer patients and patients who fulfil the criteria for genetic testing, while also providing important details about the genetic profile of Hungarian patients.

## Introduction

Cancer is among the leading causes of mortality all over the world. Over the last decade, the incidence of malignancies has been growing worldwide, resulting in an estimated 20 million new cases and 9.7 million deaths in 2022. In most types of cancer, mortality has decreased due to preventive measures, improved diagnostic tests, screening programmes and more effective treatment options [1, 2]. Unfortunately, predictions lead us to believe, that we can expect a steady increase in the number of new cases in the upcoming years [3].

In Hungary, breast cancer is the third most common malignancy after lung cancer and colorectal cancer, and the leading cause of cancer incidence in the female population, resulting in approximately 8,000 new cases and 2,000 deaths every year according to the Hungarian Cancer Registry [4].

Until recently, environmental causes were viewed as the main reason for the occurrence of malignancies. Akin to most multifactorial diseases, breast cancer is known to be associated with certain factors, that affect one’s risk to develop the disease in their lifetime. Modifiable risk factors include obesity, consumption of tobacco and alcohol, and taking hormones, while reproductive history, increasing age and a positive family history belong to non-modifiable risk factors [5–7].

In recent decades it has become apparent, that genetic variants play a considerable role in the development of breast- and ovarian cancer. While malignancies in general are usually a result of somatic mutations [8, 9], approximately 5%–10% are caused by germline mutations in moderate- or high penetrance cancer susceptibility genes [10]. These predisposing genetic traits usually follow an autosomal dominant inheritance, leading to patients having a highly elevated cumulative risk of developing different types of malignancies, and a 50% chance of passing the mutation onto their offspring [11].

Over the last few decades, diagnosis of hereditary cancer syndromes has shown promising progress. With rapid advances in molecular testing and next-generation sequencing, a great number of individuals and families have been identified, who are affected by mutations in cancer susceptibility genes. While the BRCA-related hereditary breast- and ovarian cancer (HBOC) is among the most well-known tumour syndromes, one cannot skim over the importance of other, less common predisposition genes.

Due to a lack of data on genetic spectrum of the Hungarian HBOC population, we aimed to determine the prevalence of genetic variants of HBOC-associated genes in the Hungarian patient cohort and in healthy relatives with family history of cancer; to evaluate the associations of detected variants with clinical features; to evaluate how worthwhile it is to use a larger gene panel (including other genes beside *BRCA*s) in routine genetic diagnostics of Hungarians; and to evaluate the association between familial history of cancer and familial accumulation of the genetic variants in the cohort.

## Subjects and methods

### Subjects

The examinations related to this retrospective study were carried out in the Department of Medical Genetics in the University of Pécs. The patients and relatives that were involved into this study derived from the joint genetic counselling facility of Department of Medical Genetics and Department of Oncotherapy at the University of Pécs (Oncogenetic Counselling and Family Screening Centre). Patients’ data (personal history of cancer, tumour type, and age at diagnosis) were obtained from clinical records, while the family history of cancer was obtained from interviews with the probands. People referred to the genetic counselling went through a cancer genetic risk assessment. The purpose was to identify individuals at elevated cancer risk who may benefit from genetic testing, additional screening or preventive interventions. Using clues from the personal and family history, the genetic counsellor classified individuals as average (general population), increased (moderate), or high (strong) risk patients according to the widely accepted National Comprehensive Cancer Centre (NCCN) Clinical Practice Guidelines in Oncology, which are available online for experts^1^. After risk assessment, individuals classified into the average risk level group were excluded from the study. We highlight the genetic risk stratification for HBOC in Table 1.

**TABLE 1:** Genetic risk stratification for HBOC patients.

Risk level	Personal and family history evidence
Average	No first- or second-degree relatives^a^ with breast or ovarian cancer; OR
	More distant relatives with a breast cancer diagnosis or diagnoses after age 50
Increased (moderate)	Prior history of breast cancer, lobular neoplasia (LCIS/ALH), or atypical ductal hyperplasia
	5-year risk ≥1.7% in women ≥35 years using the Gail model, or ≥20% lifetime risk defined by risk models
	Thoracic radiotherapy <30 years
	One or two first- or second-degree relatives with breast cancer diagnosis after age 50
High (strong)	Known pathogenic variant (mutation) in the family
	Somatic tumor testing suggesting a hereditary cancer syndrome
	Patient or first-degree relative diagnosed with exocrine pancreatic cancer
	Diagnosis in patient, or first- or second-degree relative with
	breast cancer at age 45 or younger
	triple negative breast cancer diagnosed at any age
	multiple primary breast cancers
	ovarian, certain prostate cancers, or male breast cancer at any age
	breast cancer or prostate cancer diagnosis at any age and Ashkenazi Jewish ancestry
	Two or more first- or second-degree relatives on same side of family with breast cancer, if at least one was diagnosed ≤ age 50
	Three or more total diagnoses of breast cancer in patient, first- or second-degree relatives on the same side of the family

^a^ First-degree relatives are parents, children, and siblings. Second-degree relatives are grandparents, grandchildren, uncles and aunts.

Blood samples were taken from individuals with increased (moderate) and high (strong) risk for cancer and they were included in the study and for further examination. We collected 370 samples from patients with any cancer type. We classified the samples according to cancer types and we assorted 205 HBOC patients for further examination. Patients with other cancer types were excluded from the current analyses. Besides, we involved 70 healthy persons with a positive family history of HBOC-related cancer, e.g., breast, ovarian, prostate or pancreatic cancer into our examination.

All individuals gave their consent to genetic testing and biobanking, which were conducted following the WMA Declaration of Helsinki - Ethical principles for medical research involving human subjects (2013) and the Hungarian genetic law (XXI/2008). The study was approved by the Regional Research Ethics Committee of the University of Pécs (8770-PTE2021) and by the Medical Research Council (IV/3831-3/2021/EKU).

### Methods

Whole peripheral blood samples were taken from all patients. DNA was extracted from the blood leukocytes using E.Z.N.A. Blood DNA Maxi Kit (Omega Biotek, Norcross, GA, United States) according to the protocol. QubitTM 2.0 Fluorometric Quantitation system (Thermo Fisher Scientific Inc., Waltham, MA United States) was used to calculate the quantity and quality of genomic DNA samples (Qubit™ 1X dsDNA HS (High Sensitivity) Assay Kits) according to the protocol. The DNA samples were deposited into the biobank of the department.

The patients were examined using Agilent SureMASTR Hereditary Cancer Kit previously, which was no longer manufactured (Agilent Technologies, Santa Clara, CA United States), thereafter we used TruSight Hereditary Cancer Kit (Illumina Inc., San Diego, CA United States). Both Kits are targeted sequencing panels. The SureMASTR Hereditary Cancer Kit contained 26 cancer-associated genes (*ATM*, *BARD1, BLM, BRCA1, BRCA2, BRIP1, CDH1, CHEK2, EPCAM (3’UTR), FAM175A, MEN1, MLH1, MRE11A, MSH2, MSH6, MUTYH, NBN, PALB2, PMS2, PTEN, RAD50, RAD51C, RAD51D, STK11, TP53,* and *XRCC2*), the TruSight Hereditary Cancer Kit designed to assess germline mutations across 113 genes (*ACD, AIP, AKT1, APC, ATM, BAP1, BARD1, BLM, BMPR1A, BRCA1, BRCA2, BRIP1, CASR, CDC73, CDH1, CDK4, CDKN1B, CDKN2A, CEBPA, CHEK2, CTRC, DDB2, DICER1, DIS3L2, EPCAM, ERCC1, ERCC2, ERCC3, ERCC4, ERCC5, FAM175A, FANCA, FANCB, FANCC, FANCD2, FANCE, FANCF, FANCG, FANCI, FANCL, FANCM, FH, FLCN, GALNT12, GATA2, GPC3, GREM1, HOXB13, KIF1B, KIT, LZTR1, MAX, MEN1, MET, MITF, MLH1, MRE11A, MSH2, MSH3, MSH6, MUTYH, NBN, NF1, NF2, NSD1, NTHL1, PALB2, PDGFRA, PHOX2B, PIK3CA, PMS2, POLD1, POLE, POT1, PRKAR1A, PTCH1, PTEN, RAD50, RAD51, RAD51B, RAD51C, RAD51D, RB1, RECQL4, RET, RHBDF2, RINT1, RUNX1, SDHA, SDHAF2, SDHB, SDHC, SDHD, SLX4, SMAD4, SMARCA4, SMARCB1, SMARCE1, SPINK1, SPRED1, STK11, SUFU, TERF2IP, TERT, TMEM127, TP53, TSC1, TSC2, VHL, WT1, XPA, XPC,* and *XRCC2*) commonly associated with hereditary predisposition to several cancers like breast, ovarian, colon, and gastric and tumour associated syndromes like tuberous sclerosis and neurofibromatosis. The SureMASTR Hereditary Cancer Kit assay uses amplicon sequencing method. The TruSight Hereditary Cancer Kit assay uses hybrid-capture chemistry integrated with Nextera Flex for Enrichment. We performed the assays according to the description of the manufacturer.

After sequencing reactions base called raw sequencing data was transformed into FASTQ format using Illumina’s software (bcl2fastq). Sequence reads of each sample were mapped to the human reference genome (GRCh37/hg19). Burrows-Wheeler Aligner (BWA-MEM) software was used for reading alignment. Duplicate read marking, local realignment around indels, base quality score recalibration and variant calling were performed using GATK algorithms (Sentieon Inc., California, United States). Variant data was annotated using a collection of tools (VcfAnno and VEP) with a variety of public variant databases including but not limited to gnomAD^2^, ClinVar^3^ and HGMD^4^. The variants detected were adapted to available public databases, depositories like Ensembl^5^, Varsome^6^, Franklin^7^; and HGMD^8^ and were classified according to the latest guidelines and interpretation criteria established by the American College of Medical Genetics and Genomics. The variants received were matched to the clinical features of the patients and the persons with positive family history.

We used PCR for validation of variants detected by NGS. The accession numbers of the gene reference sequences applied for designing methods were summarized in Supplementary Table S1 (Supplementary Material). For amplification of the region of interest, we designed specific primers (Integrated DNA Technologies Inc., Coralville, IA, United States) using the NCBI-PRIMER-BLAST tool (NCBI, Bethesda, MD, United States). The sequences of the applied oligonucleotide primers were summarized in Supplementary Table S1 (Supplementary Material).

We performed PCR reactions according to the protocol below:

Final volume was 50 μL, which contained 200 µM of each dNTP (1 μL), 0.2 mM of each primer (1–1 μL), 5 μL of reaction buffer (containing 500 mM KCl, 10 mM Tris-HCl, 14 mmol/L MgCl2, pH 9.0), 1 U of Taq polymerase (1 μL of 10 U/µL) and 1 μg extracted DNA as template (1 μL). The conditions of the polymerase chain reactions for the detection of HBOC variants were as follows: initial denaturation at 96°C followed by 35 cycles of 30 s at 95°C; 30 s at the annealing temperature; 30 s at 72°C and a final extension at 72°C. The annealing temperatures applied for certain variants are indicated in Supplementary Table S1 (Supplementary Material). The polymerase chain reactions were carried out using a SensoQuest Labcycler (SensoQuest GmBH, Göttingen, Germany).

After the amplification reaction, the products were electrophoresed through an ethidium-bromide-stained 1.5%–2% agarose gel and visualized by UVIdoc gel documentation system (UVITEC, Cambridge, UK).

The sequencing reactions were performed by Sanger sequencing on an Applied BiosystemsTM 3500 Genetic Analyzer (Thermo Fisher Scientific Inc., Waltham, MA, United States) using BigDye Terminator Chemistry. The sequence alignments were made using the Winstar genetic program (DNASTAR Inc., Madison, WI, United States).

## Results

### General characterization

We summarized the general characteristics of the study groups in Table 2. We have analysed a total of 275 samples with targeted next-generation sequencing panels of which, 205 agreed with HBOC criteria (HBOC cohort) and 70 were the sample of healthy persons with a positive family history of different cancers (non-HBOC cohort). The samples derived from non-consanguineous Hungarian people. HBOC patients’ data (age, tumour type and age at diagnosis) and non-HBOC person’s data were summarized in Table 2. Among the HBOC cases, 193 (94.1%) were females and 12 (5.85%) were males, all with a history of HBOC-related cancer. The average age of HBOC cases was 56.0 ± 15.0 years. The lowest age discovered was 21 years while the highest was 84 years. The average age at diagnosis for HBOC cases was 46.3 ± 11.7 years (range = 15–78 years). Among HBOC patients, 51.2% had their primary cancer diagnosis at age 45 years or younger. The types of cancers identified within the HBOC group were breast cancer (n = 179; 87.3%), ovarian cancer (n = 16; 7.81%), prostate cancer (n = 6; 2.93%), uterus cancer (n = 3; 1.47%) and endocervix cancer (n = 1; 0.49%).

**TABLE 2:** Patient cohorts’ characteristics.

Cohort of HBOC patients^a^	
Gender n (%)	
Female	193 (94.1%)
Male	12 (5.85%)
total	205
Age (years)	
Average ±SD	56.0 ± 15.0
Min–max	21–84
Age at diagnosis (years)	
Average ±SD	46.3 ± 11.7
Min–max	15–78
≤45	n = 105 (51.2%)
45–60	n = 70 (34.2%)
>60	n = 30 (14.6%)
Tumour types n (%)	
Breast cancer	179 (87.3%)
Ovarian cancer	16 (7.81%)
Uterus cancer	3 (1.47%)
Prostate cancer	6 (2.93%)
Endocervix	1 (0.49%)
Cohort of non-HBOC persons^b^	
Gender n (%)	
Female	60 (85.7%)
Male	10 (14.3%)
total	70
Age (years)	
Average ±SD	49.0 ± 14.0
Min–max	20–81

^a^ HBOC, cohort: hereditary breast and ovarian cancer.

^b^ non-HBOC: healthy persons with hereditary cancer relatives.

Among the healthy non-HBOC cases, there were 60 (85.7%) females and 10 (14.3%) males, all without personal breast and ovarian cancer history but with a positive family history of any cancer. The average age of non-HBOC cases was 49.0 ± 14.0 years, ranging from 20 to 81 years.

In Table 3, we summarized the distribution of genetic variants in all cohorts and the age at diagnosis in the HBOC cohort. The average age at diagnosis in the carrier HBOC group was 44.1 ± 10.9 years, where 25 years was the minimum and 72 years was the maximum age at diagnosis. The average age at diagnosis in non-carriers was 47.3 ± 11.9 years. The minimum age at diagnosis was 15 years; however, the maximum age at diagnosis was 78 years. There was no significant difference in the age at diagnosis between carrier and non-carrier HBOC groups.

**TABLE 3:** Distribution of carriers and non-carriers in the cohorts.

			Age at diagnosis	
		Number of cases n (%)	Average ±SD (years)	Min–max (years)
HBOC patients (n = 205)	Variant carriers	62 (29.8)	44.1 ± 10.9	25–72
	Non-carriers	143 (70.2)	47.3 ± 11.9	15–78
non-HBOC persons (n = 70)	Variant carriers	20 (28.6)	-	-
	Non-carriers	50 (71.4)	-	-
All participants (n = 275)	Variant carriers	82 (29.8)	-	-
	Non-carriers	193 (70.2)	-	-

### Mutational profile of HBOC and non-HBOC study groups

The number of cases of HBOC patients carrying any genetic variants were 61, which is 29.8% of all HBOC patients tested. In the non-HBOC study group, 20 individuals were found carrying any genetic variant in association of cancer predisposition, which is 28.6% of the tested non-HBOC persons. The total number of all variant carriers was 82 cases, which is the 29.8% of all cases.

The complete record of the positive results of different tumour types is summarized in Table 4. The table shows the genes in which we detected variants and it contains the most prevalent variants, as well. Breast cancer was diagnosed in most of our cases (179/205; 87.3%), of which we identified genetic variants in 51 cases, representing 28.5% of the breast cancer patients: twelve *BRCA2* (6.70%), eight *CHEK2* (4.47%), eight *BRCA1* (4.47%), seven *ATM* (3.91%), three *PALB2* (1.68%), and 2-2 variants in *BARD1, FANCI* and *RAD51C* genes (1.12%), and 1-1 variant in *APC, BLM, CDH1, ERCC2, KIT, MSH6,, PMS2, RAD50, RAD51B,* and *RET* genes (0.56%). Besides, 9 out of 16 ovarian cancer cases (56.3%) harboured genetic variants: six *BRCA1* (37.5%), two *RAD51C* (12.5%) and in one case *BRCA2* (6.25%). The most prevalent variant was *BRCA1* c.5266dupC in the cohorts. Of the six prostate cancer cases, only one carried a genetic variant in the oncogenes (16.7%). From the three cases diagnosed with uterus cancer, only one case revealed positivity, where a pathogenic variant was found in the *BRCA1* gene. We could not detect any genetic alteration in the patients diagnosed with endocervix cancer by oncogenetic testing.

**TABLE 4:** Carriers of pathogenic/likely pathogenic variants and variants of uncertain significance (VUS) according to tumour types in the HBOC cohort.

Tumour types	Gene Variant detected	All carriers n (%)	Carriers of p/LP n (%)	Carriers of VUS n (%)
Breast cancer (n = 179)		51 (28.5)	34 (19.0)	17 (9.50)
	*BRCA2*	12 (6.70)	9 (5.03)	3 (1.68)
	*BRCA1*	8 (4.47)	8 (4.47)	0
	*BRCA1 c.5266dupC*	3 (1.68)		
	*CHEK2*	8 (4.47)	7 (3.91)	1 (0.56)
	*CHEK2 c.599T>C*	3 (1.68)		
	*ATM*	7 (3.91)	4 (2.23)	3 (1.68)
	*PALB2*	3 (1.68)	1 (0.56)	2 (1.12)
	*BARD1*	2 (1.12)	1 (0.56)	1 (0.56)
	*FANCI*	2 (1.12)	1 (0.56)	1 (0.56)
	*RAD51C*	2 (1.12)	1 (0.56)	1 (0.56)
	*APC*	1 (0.56)	0	1 (0.56)
	*BLM*	1 (0.56)	1 (0.56)	0
	*CDH1*	1 (0.56)	1 (0.56)	0
	*ERCC2*	1 (0.56)	1 (0.56)	0
	*KIT*	1 (0.56)	0	1 (0.56)
	*MSH6*	1 (0.56)	0	1 (0.56)
	*PMS2*	1 (0.56)	0	1 (0.56)
	*RAD50*	1 (0.56)	0	1 (0.56)
	*RAD51B*	1 (0.56)	0	1 (0.56)
	*RET*	1 (0.56)	0	1 (0.56)
Ovarian cancer (n = 16)		9 (56.3)	7 (43.8)	2 (12.5)
	*BRCA1*	6 (37.5)	5 (31.3)	1 (6.25)
	*BRCA1* c.5266dupC	3 (18.8)		
	*RAD51C*	2 (12.5)	1 (6.25)	1 (6.25)
	*BRCA2*	1 (6.25)	1 (6.25)	0
Prostate cancer (n = 6)	*CHEK2*	1 (16.7)	1 (16.7)	0
Uterus cancer (n = 3)	*BRCA1*	1 (33.3)	1 (33.3)	0
Endocervix (n = 1)		0	0	0

The identified genetic variants in the HBOC and non-HBOC groups are listed in Supplementary Table S2 (Supplementary Material). The table contains the clinical significance of the variants, which were predicted by the ClinVar database and Franklin interpretation tool. The clinical significance of most of the variants can be determined unanimously based on the data from ClinVar and Franklin. In some cases, there were discrepancies between the interpretation of ClinVar and Franklin (pathogenic-likely pathogenic, pathogenic-VUS). Based on the analysis, Franklin proved to be more permissive in most cases. In the ClinVar database, no records were found for the *CDH1* c.2499delT variant; however, after classification according to the ACMG guidelines, we interpreted it as a likely pathogenic/pathogenic variant, as was predicted by the Franklin prediction tool.

We identified pathogenic/likely pathogenic and VUS genetic variants in *APC, ATM*, *BARD1*, *BLM, BRCA1*, *BRCA2*, *CHEK2*, *CDH1*, *ERCC2, FANCI, KIT*, *MSH6*, *PALB2*, *PMS2*, *RAD50, RAD51B, RAD51C,* and *RET* in the HBOC group. The non-HBOC patients harbour pathogenic/likely pathogenic and VUS genetic variants in *BRCA1*, *BRCA2*, *CHEK2*, *EPCAM, ERCC3, FANCD2, MSH6*, *MUTYH, PALB2*, *POLE, PTCH1,* and *RAD51C.* Genetic variants in *BARD1*, *CDH1*, *PMS2*, *KIT* and *RET* genes were identified in HBOC patients only. Variants in *MUTYH*, *EPCAM*, *FANCD2*, *POLE*, *ERCC3,* and *PTCH1* were reported in the non-HBOC persons only. Overall, we identified 67 unique pathogenic/likely pathogenic variants and VUS carried by 82 individuals: 35 missense (52.2%), 15 frameshift (22.4%), 10 nonsense (14.9%), 2 synonymous (2.99%), 4 splice site variants (5.97%) and one three basepair deletion (1.49%), which cause one amino acid deletion, but not frameshift.

For *BRCA2*, 16 distinct variants in the association of cancer were identified in 17 individuals. The majority of the variants had pathogenic or likely pathogenic clinical significance. Only two variants were VUS (c.1012 G>A; c.3042 T>G). The most prevalent variant was the c.1408dupG, which were detected in two breast cancer cases, representing 2.98% of all variants detected.

For *BRCA1*, 10 different variants in association of cancer were identified in 17 individuals. Almost all variants were predicted to be pathogenic, and only one was predicted to be VUS (c.734 A>T). In the case of c.845 C>T, Franklin predicted it as VUS; however, it was mentioned as pathogenic in ClinVar. One of the variants (c.5266dupC) was present in seven samples, representing approximately 10.4% of all variants detected.

*CHEK2* is the third gene, in which we most frequently identified variants in association with cancer. We detected 6 different variants in 11 individuals, from which five were pathogenic/likely pathogenic, one was VUS (c.1039 A>G) and one had conflicting interpretations of pathogenicity (c.1684 C>G; ClinVar: pathogenic/likely pathogenic, Franklin: VUS). One of the variants (c.599 T>C) was present in six samples, representing approximately 8.96% of all variants detected.

For *ATM* gene, 7 distinct variants in the association of cancer were identified in 7 individuals, from which three were pathogenic/likely pathogenic and four were VUS according to ClinVar and Franklin.

We compared the numbers of detected cases and allele frequencies of the pathogenic/likely pathogenic variants for each gene in the study groups, which are summarized in Table 5. Pathogenic/likely pathogenic variants of *BRCA1* and *BRCA2* showed similar allele frequencies, which shared the highest frequencies among the genes in the HBOC cohort. In the non-HBOC group, *BRCA1* showed the highest frequency. Additionally, we assessed the allele frequencies of the genes in the gnomAD non-cancer cohort (n = 134 187), which showed significant differences in *BRCA1, BRCA2, CHEK2, MUTYH, MSH6, POLE, and ERCC3* genes in comparison to our non-HBOC groups.

**TABLE 5:** Detection ratio of *BRCA1*, *BRCA2*, *PALB2*, *ATM*, *CHEK2*, *MUTYH*, *MSH6*, *RAD51C*, *BARD1*, *CDH1*, *POLE and ERCC3* P/LP variants in the study groups and gnomAD non-cancer population.

Gene name	HBOC cohort (n = 205)		Non-HBOC cohort (n = 70)		All participants (n = 275)		GnomAD non-cancer cohort (n = 134 187)	
	Detected cases	Allele frequency	Detected cases	Allele frequency	Detected cases	Allele frequency	Detected cases	Allele frequency
*BRCA1*	13	0.03170	3	0.02143^a^	16	0.02909	295	0.00110
*BRCA2*	13	0.03170	2	0.01429^a^	15	0.02727	411	0.00153
*PALB2*	1	0.00244	0	0	1	0.00182	200	0.00075
*ATM*	3	0.00730	0	0	3	0.00545	428	0.00160
*CHEK2*	8	0.01950	1	0.00714^a^	9	0.01636	207	0.00077
*MUTYH*	0	0	1	0.01429^a^	1	0.00182	1,536	0.00572
*MSH6*	0	0	2	0.01429^a^	2	0.00363	119	0.00044
*RAD51C*	1	0.00244	0	0	1	0.00182	118	0.00044
*BARD1*	1	0.00244	0	0	1	0.00182	84	0.00031
*CDH1*	1	0.00244	0	0	1	0.00182	9	0.00003
*POLE*	0	0	1	0.00714^a^	1	0.00182	48	0.00018
*ERCC3*	0	0	1	0.00714^a^	1	0.00182	247	0.00092
*FANCI*	1	0.00244	0	0	1	0.00182		
*ERCC2*	1	0.00244	0	0	1	0.00182		
*BLM*	1		0	0	1	0.00182		

All individuals carried the variants in heterozygous form.

^a^ Chi-square tests were used for comparison of the allele frequencies between GnomAD, Non-Cancer Group and our non-HBOC, group.

Remarkably, *BRCA1* and *BRCA2* are the genes that explain almost half of the positive cases (17 + 17/82; 41.5%). *BRCA1* and *BRCA2* were the genes with the highest rate of pathogenic/likely pathogenic variants. Three patients diagnosed with breast cancer in the HBOC group harboured two genetic variants concomitantly (double heterozygotes; DH): 1. patient: *BRCA1* c.5266dupC het, p.Gln1756Profs*74 and *BLM* c.1642C>T het, p.Gln548Ter, which are known pathogenic variants; the patient was diagnosed with Grade 3 triple negative, invasive ductal carcinoma at the age of 32 years. 2. patient: *BRCA2* c.7976G>A het, p.Arg2659Lys and *RAD50* c.287T>C het, p.Val96Ala, which are pathogenic and VUS, respectively; the patient has been receiving treatment for Grade 3 triple negative, invasive ductal carcinoma as well, which was diagnosed at the age of 29 years. 3. patient: *BRCA2* c.3042T>G het, p.Asn1014Lys and *ATM* c.5890A>G het, p.Lys1964Glu, which are VUSs. The patient was first diagnosed with Grade 3 triple negative invasive ductal carcinoma at the age of 35 years, and a contralateral Grade 3 triple negative ductal breast cancer at the age of 44 years. Though there were suspicions for causality based on the patient’s phenotype, the initial classification of these variants remained unchanged upon reevaluation.

Besides, we identified other variants with benign clinical significance by next-generation testing, which were not confirmed by Sanger sequencing and they are not the part of this study.

## Discussion

Over the last decade, the spreading of new techniques such as NGS that allows the simultaneous analysis of multiple genes in panels as well as whole exome/genome are more cost-effective and efficient compared to Sanger sequencing. The use of these techniques probably contributes to faster recognition of the genetic cause of rare or ultra-rare disorders and to broadening the genetic screening of certain disorders, like cancers, where rapid diagnosis is extremely important for further care and the selection of appropriate therapy.

In our work, NGS allows us to determine the prevalence of genetic variants of HBOC-associated genes in the Hungarian patient cohort. Such data were not available for the Hungarian population, so far. We focused on the prevalence of genetic variants, predominantly pathogenic/likely pathogenic variants in cancer susceptibility genes in a Hungarian patient cohort selected from gathering increased (moderate) and high (strong) risk criteria for HBOC and a healthy individual’s cohort, whose family history was positive for any cancer.

Pathogenic/likely pathogenic variants and VUSs were found in almost 50% of all individuals in HBOC as well as in non-HBOC groups. We found variants in the following genes: *APC*, *ATM*, *BARD1*, *BLM*, *BRCA1* and *BRCA2, CDH1, CHEK2, EPCAM, ERCC3, FANCD2, KIT, MSH6, MUTYH, PALB2, PMS2, POLE, PTCH1, RAD, RAD51B, RAD51C,* and *RET.* These genes are involved in repairing DNA damage and maintaining the stability of the cell’s genetic material [12]. Some of these genes play important roles particularly in the homologous recombination-, base excision- and mismatch repair pathway (HRR, BER, MMR), which are important for maintaining genomic stability [13–15].

It is well-established that due to pathogenic germline variants detected in these genes, cells can grow uncontrolled and carrying these variants can confer predisposition for the development of hereditary cancer syndromes, which affects 5% of all HBOC cases. In the Hungarian HBOC group, *BRCA1* and *BRCA2* genes proved to be highly penetrant genes with their pathogenic detection rate of 6.34% (13/205, each). While *CHEK2* and *ATM* have moderate penetrance in HBOC (3.90%, 8/205; 1.46%, 3/205, respectively); in turn, the *PALB2* gene has a lower detection rate of 0.48% (1/205). These results are consistent with others’ findings in European populations with HBOC [16, 17]. In the non-HBOC group, we obtained similar results regarding the *BRCA1* gene (4.29%, 3/70); however, there are differences in the prevalence of other genes. These differences can result from the fact, that in this group there were healthy people, who have a positive family history with a wide range of tumour spectrum including colorectal, pancreatic or gastric cancers, which have been associated with other genes such as *MUTYH* and *MSH6*.

Furthermore, the HBOC group was divided into subgroups according to the type of cancer: breast, ovarian, prostate, uterus and endocervix subgroups, and we evaluated the prevalence of genetic variants in each group. In the breast cancer subgroup, variants in *BRCA2* and *BRCA1* were the most frequently detected. In the ovarian cancer subgroup, *BRCA1* variants, mainly c.5266dupC was found. Besides, rare variants in other genes like *ATM*, *CHEK2*, *CDH1*, *PALB2*, *RAD50,* and *RAD51C* have been detected mainly in the breast cancer subgroup. Most of these variants detected are variants of uncertain significance (VUS). In clinical practice, VUSs represent a challenge, because they must be indicated on medical results; however, - according to ACMG recommendations - VUSs are not used to guide clinical management until the clarification of their exact role [18]. Almost all of the VUSs detected in the study are located within important regions of the genes or functional domains of the proteins and could affect biochemical properties and biological functions. Even though we cannot determine their clinical significance with current evidence, functional studies on these variants would be necessary to explore their phenotype consequences. In our study, these variants of uncertain significance account for high percentage of the positive cases, furthermore we detected a surprising number of pathogenic variants in moderate-, and low penetrant genes. There are differences among European countries in the judgement of involving low penetrance genes in the first-line genetic HBOC screening. In some countries (e.g., Austria, Estonia, Finland, Poland), the hotspot and/or founder variants of BRCA or HBOC genes are tested first. In other countries like Finland and the Netherland, WES is executed and evaluated as an oncogenetic panel. However, it may vary among laboratories within a given country. Our findings highlight the possible benefits of including other genes besides *BRCA*s in routine genetic testing. However, incorporation testing of several genes into multigene (*PMS2, RET, KIT*) for which the evidence of risk-association is weak or absent can result in information that might not be clinically relevant to the affected patients and their families [19, 20].

In the Hungarian cohorts, the c.5266dupC in the *BRCA1* gene was detected most frequently (7/82, 8.54%). This variant is an insertion of one cytosine resulting in a frameshift and the creation of a novel translational termination codon after 74 amino acid residues. It is predicted to encode a truncated non-functional protein due to nonsense-mediated mRNA decay, which is a commonly known mechanism for disease. Functional studies have shown activity of the protein is significantly impaired by this variant [21]. This mutation has been described in individuals with HBOC, including individuals with male breast cancer, pancreatic cancer, and prostate cancer [22–27], and has been shown to be a founder mutation in several ethnic groups [28]. The high prevalence of this variant in our cohort and the fact that this variant has been reported several times in the literature state the probability of being a founder mutation of our population or maybe for a specific ethnicity; however, we did not collect information about the patient’s origin. To confirm this hypothesis, further studies are required.

To evaluate the association between familial history of cancer and accumulation of the genetic variants, we assessed allele frequency data between our non-HBOC group and the gnomAD non-cancer non-Finnish European population as a group of healthy (non-cancer) individuals without any family history of cancer [29]. The findings revealed significant differences in allele frequencies between the two study groups, so the healthy persons with elevated risk for cancer (because of positive family history) carry the pathogenic variants in a higher proportion than people in the average population. Generally, indications of testing for high-penetrance susceptibility genes include a personal history of cancer at a young age, triple-negative or male breast cancer, epithelial ovarian cancer, multiple primary malignancies, or a positive family anamnesis. Based on these facts, genetic screening of predisposing genes in healthy but elevated-risk persons would be necessary to prevent developing tumours. After screening, healthy carriers can be easily managed and can be involved in surveillance programs and preventive follow-up care. Recognizing at-risk patients who carry the aforementioned genetic variants has important consequences on an individual level and on a larger scale as well. Affected individuals, who are already undergoing treatment can receive targeted therapy based on their genetic results, which usually leads to better outcomes and higher probability of long-term survival. Since their risk for developing other malignancies is also elevated, a positive genetic result also warrants a multidisciplinary approach to their surveillance. Identification of healthy carriers offers patients an opportunity to attend regular screening from a young age and the possibility of certain, primary preventive measures, including chemoprevention and risk-reducing surgery [30–33]. These surveillance strategies lead to earlier detection and better prognosis, reducing premature mortality and prolonged disability, while also easing the strain of costs on the healthcare system.

Besides, we detected a novel germline variant (*CDH1* c.2499delT), which is not yet recorded in variant databases. We interpreted and classified this sequence variant according to the standards and guidelines published by the American College of Medical Genetics and Genomics and Association for Molecular Pathology (ACMG-AMP) [34, 35]. This variant is predicted to cause loss of normal protein function either through protein truncation or nonsense-mediated mRNA decay resulting in an absent or non-functional protein product (PVS1). Other frameshift variants in the nearby region have been reported in individuals with hereditary cancer-predisposing syndrome, and Familial cancer of breast, loss-of-function of *CDH1* is a well-established mechanism leading to the disorder. This variant has not been observed in the large reference population cohorts of the Genome Aggregation Database (gnomAD, n > 120 000 exomes and n > 15 000 genomes) (PM2). To our knowledge, the variant has not been published in the relevant medical literature or reported in disease-related variation databases such as HGMD or ClinVar. To assess the impact of the missense change, we applied a combination of some of the most widely used “*in silico*” computational prediction tools like PolyPhen, Provean and MutationTaster [36]. These algorithms support obvious evidence that the c.2499delT variant is a protein damaging, disease-causing variant (PP3). To our best knowledge, there are no “*in vitro*” functional studies which investigate the damaging effect of this variant on the gene product (PS3). The classification of this *CDH1* variant provides strong evidence of pathogenicity. The patient was diagnosed with metastasizing lobular breast carcinoma at the age of 48 years. The histological subtype of her tumour suggests furthermore, that there is a cause-effect relation between her illness and the detected germline variant.

Double heterozygosity (DH) in a high-, or moderate penetrance gene is a rare event; however, its number is likely to increase due to the use of NGS. In our study, we report three breast cancer cases (3/205; 1.46%) with (1) two VUSs in *BRCA2* and *ATM* genes; (2) one pathogenic variant in the *BRCA2* gene and one VUS in the *RAD50* gene; (3) two pathogenic variant in *BRCA1* gene. Some studies showed no significant differences in risk for HBOC in DH carriers when compared with those carrying only one germline variant [37, 38]. Others suggest that this genotype is not associated with early disease onset or a more severe phenotype [38]. In regards to our patients, all three were diagnosed with high grade, triple negative ductal carcinoma at a very young age. However, ethnic differences have been described as Caucasian DH females might develop breast cancer at an earlier age and have more severe disease in contrast to e.g., Ashkenazi Jewish females [37]. However, despite the little information about phenotype of DH carriers, we should not ignore their assumed risk modifier role and their possible cumulative impact on the development of the phenotype supporting a polygenic risk that should be considered in risk prediction in genetic counselling. Furthermore, DHs should be involved in more intensive surveillance programs/follow-up care and may need more severe prophylactic surgery [39].

The limitation of our study is the small number of individuals in certain groups with cancer types, which does not allow us to make profound genotype-phenotype associations. Besides, associations assumed in this study can be reliably assessed with information on other extrinsic contributing factors like smoking, alcohol consumption and dietary habits of the patients. These should be evaluated on a larger cohort of cancer patients.

## Conclusion

The prevalence of hereditary mutations in breast, ovarian, endometrial and prostate cancer highlights the significance of genetic counselling and molecular genetic testing concerning these malignancies. Our study demonstrates a considerable percentage of pathogenic mutation carriers, emphasizing the benefits of molecular screening tests. The expansions of the diagnostic field revealed many other pathogenic variants associated with HBOC cancer that could have been missed if only *BRCA1* and *BRCA2* genes had been analysed. In this context, the appearance of next-generation sequencing methods has great potential. Thanks to its expansion, many new variants have been discovered in previously undiagnosed patients. Besides, comprehensive genetic diagnostics have long-term clinical benefits like increased patient cure rates, better patient surveillance, reduced cancer mortality rates, more effective treatments, and rapid recognition of high-risk families to reduce the risk of developing cancer. However, new challenges are generated in emerging next-generation sequencing like the interpretation of the huge amount of data derived from NGS, which highlights the need for highly skilled professionals in genetic counselling and molecular diagnostics with close cooperation of a multidisciplinary team.

Our results provide important new information to ethnic-specific cancer risk profiling and to determining even polygenic risk scores for HBOC, which can increase the sensitivity and specificity of cancer diagnostics as well as lowering the cost. However, several rare variants were identified with uncertain clinical significance, evaluation of the biological impact of these variants, further functional assays or robust segregation studies are needed.

## Data Availability

The datasets presented in this study can be found in online repositories. The names of the repository/repositories and accession number(s) can be found in the article/Supplementary Material.

## Footnotes

1
www.nccn.org

2
https://gnomad.broadinstitute.org/

3
https://www.ncbi.nlm.nih.gov/clinvar/

4
https://www.hgmd.cf.ac.uk/ac/index.php

5
https://ensemble.org

6
https://varsome.com/

7
https://franklin.genoox.com/clinical-db/home

8
http://www.hgmd.cf.ac.uk/ac/index.php
