# Supplementary material for: Molecular genetic investigation of hereditary breast and ovarian cancer patients in the Southern Transdanubian region: widening the mutation spectrum and searching for new pathogenic variants using next-generation methods
Source: Pathol Oncol Res. 2024 Aug 1;30:1611813. doi: 10.3389/pore.2024.1611813 (PMC11324426; doi:10.3389/pore.2024.1611813)
Supplement: Supplementary file 1 [file DataSheet1.docx]

| **Table S1.** Methods for validation of the genetic variants found in the cohorts. | | | | |
| --- | --- | --- | --- | --- |
|  |  |  |  |  |
| **Gene** | **Variant** | **5’-Forward Primer-3’** | **5’-Reverse Primer-3’** | **Annealing Temperature for PCR (°C)** |
| *ATM*  (NM_002519.3) | c.1550 T>C | CTACCCCAACAGCGACATGG | TCGGCCAAACAAGAAAAGCATC | 57 |
|  | c.2250 G>A | CAGAAACTCTTGTCCGGTGT | ATAAAAGAGAAAGGGTTAACCTGC | 60 |
|  | c.3154-2 A>G | GGCAAGGTGAGTATGTTGGC | GCCATCTGCAGCATTCCAAAT | 55 |
|  | c.3802delG | GTGCTACTGAACAAGGTCCCA | ACATAAAAATTGAAGCCATACCTGT | 60 |
|  | c.8228 C/T | GGGAGACTGTCAAGAGGTGC | ACTCCTGGTCCAAATAATGGCA | 57 |
| *BARD1*  (NM_000465.4) | c.1339 C>G | GCACATGATCTTTGGCCAGT | GAGTATATGTGGCAGAGGATGA | 55 |
|  | c.1690 C>T | GAAGAAATGAGATGCCCTGGGT | TGTTCTGAAGACAGCCCACTG | 60 |
| *BRCA1*  (NM_007294.4) | c.68_69delAG | GACGTTGTCATTAGTTCTTTGG | GGTCAATTCTGTTCATTTGC | 55 |
|  | c.181 T>G | CTCTTAAGGGCAGTTGTGAG | ATGGTTTTATAGGAACGCTATG | 55 |
|  | c.734 A>T, c.845 C>T | CCACCTCCAAGGTGTATGA | TGTTATGTTGGCTCCTTGCT | 55 |
|  | c.2329delT | GACAGCGATACTTTCCCAGA | TGGAACAACCATGGATTAGTC | 55 |
|  | c.5137 G>C, c.5251 C>T, c.5266dupC | AGCTGTGTGCTAGAGGTAACTC | GTGGTTTTATGCAGCAGATG | 55 |
|  | c.1529 C/G, c.1687 C>T | CAAGAGCGTCCCCTCACA | GCGCATGAATATGCCTGGT | 55 |
| *BRCA2*  (NM_000059.4) | c.658_659delGT | GCCATATCTTACCACCTTGTGA | AGGTTTAGAGACTTTCTCAAAGGC | 55 |
|  | c.1012G>A | CAGCATCTTGAATCTCATACAG | GTGGATATTAAACCTGCATTCTTCAA | 56 |
|  | c.1408dupG | TATGTCCAAATTTAATTGATAAT | AAACACAGAAGGAATCGTCATC | 55 |
|  | c.1902+2 T>C | TCTAGAGGCAAAGAATCATA | TCATTGTCTGAGAAAAGTTC | 55 |
|  | c.3042T>G | ACAAATGGGCAGGACTCTTAGG | CCTGCTTGGAAAATAACATCTG | 57 |
|  | c.5645 C>A | AAGACATATTTACAGACAGT | TGAAGCTTCCCTATACTACAT | 55 |
|  | c.6644_6647delACTC | TTCAACAAGACAAACAACAGT | TGTCAGTTCATCATCTTCCATAAA | 55 |
|  | c.6957delA | GCATCCGTTACATTCACTGAAA | ACGGGAAGTGTTAACTTCTTAACG | 55 |
|  | c.7975 A>G | CAGAGAATAGTTGTAGTTGTTGAA | GCAACATTTTGACATGGAAGTCAC | 55 |
|  | c.8249_8251del | GCAGATACCCAAAAAGTGGC | TCTGGACCTCCCAAAAACTG | 55 |
|  | c.8755-1 G>A | AACCACACCCTTAAGATGAGC | GGGCATTAGTAGTGGATTTTGC | 55 |
|  | c.9097dupA | ATATGACGTGTCTGCTCCAC | AGTCTTACAAAATGAAGCGG | 55 |
|  | c.9117 G>A | ACTTCTTCCATTGCATCTTTCTCA | AAAACAAAACAAAAATTCAACATA | 55 |
| *CDH1*  (NM_004360.5) | c.2499delT | CCTTTCTGGGTGGAAATGCAG | TCAGGGAGCTCAGACTAGCA | 57 |

| \| **Table S1. (Continued)** \| \| \| \| \| \| --- \| --- \| --- \| --- \| --- \| \|  \|  \|  \|  \|  \| \| **Gene** \| **Variant** \| **5’-Forward Primer-3’** \| **5’-Reverse Primer-3’** \| **Annealing Temperature for PCR (°C)** \| \| *CHEK2*  (NM_007194.4) \| c.277delT \| CTCTACCAGCACGATGCCAA \| GCATAAGGCGTCAATCCACAC \| 55 \| \| c.599 T>C \| CAGGGTAGGTAATGAATACCCATGT \| TTCAAAGGACGGCGTTTTCC \| 55 \| \| c.1031delT, c.1039 A>G \| ACCTAGCCTTCATTCCCAGGTAGC \| CCAGGATGAGAAAGGCAAGCCTAC \| 60 \| \| c.1100del \| TGTCTTCTGTCCAAGTGCGT \| CCACGGTCCCTCGATTTCT \| 60 \| \| c.1684 C/G \| CACAAAGGTTGTCTTCCCGC \| ACGGAGTTCACAACACAGCA \| 56 \| \| *EPCAM*  (NM_002354.3) \| c.577 G>A \| CCCTGAGCTGTCTGCTTAAAGA \| AGATCCGAAACTGTTGCTCCA \| 56 \| \| *ERCC3*  (NM_000122.1) \| c.325 C>T \| CTGTGGTGTTGGGCAGCTTA \| ATTAGCAGGGCAGGTGGAAT \| 55 \| \| *FANCD2*  (NM_001018115.1) \| c.2803_2804delAT \| CCCCATAGGAGTTCACAGGG \| TCTCCTCAGTGTCACAGTGTTC \| 55 \| \| *KIT*  (NM_000222.3) \| c.757-9 A/G \| GCAATGTGTTCGCTCTCAGT \| CTGCCAGTAATTTAAACATTCCCA \| 55 \| \| *MSH6*  (NM_000179.2) \| c.2315G/A \| CCCTCTCTGCTCTAGGTGGT \| TATAGCCCTGCTGTCTGGGT \| 57 \| \| c.3261dupC \| GCGCAACAGAATTGGCTGTA \| GCTGGCAAACAGCACTACTT \| 56 \| \| c.3961 A/G \| GAATGTGAAGACCCCAGCCA \| TGTTTCTTTGAAACTTAAGGTCAGT \| 55 \| \| *MUTYH*  (NM_001048174.2) \| c.1187 G>A \| CACTCTCTAGGTTGGCCCCT \| CAACATCCTTGGCTATTCCGC \| 56 \| \| *PALB2*  (NM_024675.4) \| c.1250 C>A \| AGCGGTAGTAGTCAGCACCT \| CCTCCACGGCTACTTTCCTC \| 57 \| \| c.2167_2168delAT \| CAAACTCGCAAAGCCAGCAT \| CCAATGCGCAAGCAAGTCA \| 55 \| \| c.3217 G>T \| AAACACTGGGAACCGGAAGC \| CAGTTATGCACACTTGCCTGC \| 57 \| \| *PMS2*  (NM_000535.5) \| c.2206 G>A \| GACCTCAGACTCTCAACTTAACTGC \| TCACCATGTTAGCCAGGCTGGT \| 55 \| \| *PTCH1*  (NM_000264.5) \| c.901 G>A \| CGCAGCCGTGTTACTTTACG \| GCTCACTGCTGGTACTCACT \| 55 \| \| *RAD51C*  (NM_058216.3) \| c.323 A>G \| AGCACTGGAACTTCTTGAGCA \| CGCAGAAACTTCCTGTTTAACCT \| 56 \| \| c.502 A>T \|  \|  \|  \| \| c.862 A>G \| TGTCCAGGCTGGAGTGAAGTG \| AATGCAGGAACAAGCAAGGC \| 59 \| \| *RET*  (NM_020975.6) \| c.2348 A>C \| CGAGTGAGCTGCGAGACCTGC \| GGCAGTGTCACACCAGAGACC \| 55 \|   **Table S2.** Identified pathogenic/likely pathogenic genetic variants and variants of uncertain significance. | | | | | | | | | | | | | | | | | | | |
| --- | --- | --- | --- | --- | --- | --- | --- | --- | --- | --- | --- | --- | --- | --- | --- | --- | --- | --- | --- | --- | --- | --- | --- | --- | --- | --- | --- | --- | --- | --- | --- | --- | --- | --- | --- | --- | --- | --- | --- | --- | --- | --- | --- | --- | --- | --- | --- | --- | --- | --- | --- | --- | --- | --- | --- | --- | --- | --- | --- | --- | --- | --- | --- | --- | --- | --- | --- | --- | --- | --- | --- | --- | --- | --- | --- | --- | --- | --- | --- | --- | --- | --- | --- | --- | --- | --- | --- | --- | --- | --- | --- | --- | --- | --- | --- | --- | --- | --- | --- | --- | --- | --- | --- | --- | --- | --- | --- | --- | --- | --- | --- | --- | --- | --- | --- | --- | --- | --- | --- | --- | --- | --- | --- | --- | --- | --- | --- | --- | --- | --- | --- | --- | --- | --- |
| **Gene name** | **Sequence Variant Nomenclature on the cDNA level according to HGVS*** | | **Sequence Variant Nomenclature on the amino acid level according to HGVS*** | | | **Variant Type, Molecular Consequence** | | | **Variant rsID (if exist)** | | **Nr of Patients Carrying the Variant, Cohort** | | | | | **ClinVar Interpretation** | | **Franklin Interpretation** | |
| *BRCA1* | c.68_69delAG | | p.Glu23ValfsTer17 | | | Deletion, frameshift | | | rs80357914 | | 1, HBOC | | | | | Pathogenic | | Pathogenic | |
|  | c.181T>G | | p.Cys61Gly | | | SNV, missense | | | rs28897672 | | 2, HBOC | | | | | Pathogenic | | Pathogenic | |
|  | c.734A>T | | p.Asp245Val | | | SNV, missense | | | rs80356865 | | 1, non-HBOC | | | | | VUS | | VUS | |
|  | c.845C>T | | p.Ser282Leu | | | SNV, missense | | | rs786203027 | | 1, HBOC | | | | | Pathogenic | | VUS | |
|  | c.1529C>G | | p.Ser510Ter | | | SNV, nonsense | | | rs80357427 | | 1, non-HBOC | | | | | Pathogenic | | Pathogenic | |
|  | c.1687C>T | | p.Gln563Ter | | | SNV, nonsense | | | rs80356898 | | 1, HBOC | | | | | Pathogenic | | Pathogenic | |
|  | c.2329delT | | p.Tyr777fsTer15 | | | Deletion, frameshift | | | rs80357725 | | 1, non-HBOC | | | | | Pathogenic | | Pathogenic | |
|  | c.5137G>C | | p.Asp1713His | | | SNV, missense | | | rs80187739 | | 1, HBOC | | | | | Pathogenic | | Likely Pathogenic | |
|  | c.5251C>T | | p.Arg1751Ter | | | SNV, nonsense | | | rs80357123 | | 1, HBOC | | | | | Pathogenic | | Pathogenic | |
|  | c.5266dupC | | p.Gln1756ProfsTer74 | | | Duplication, frameshift | | | rs80357906 | | 6, 1 (HBOC, non-HBOC) | | | | | Pathogenic | | Pathogenic | |
| *CHEK2* | c.277delT | | p.Trp93GlyfsTer17 | | | Deletion, frameshift | | | rs786203458 | | 1, HBOC | | | | | Pathogenic | | Pathogenic | |
|  | c.599T>C | | p.Ile200Thr | | | SNV, missense | | | rs17879961 | | 4, 2 (HBOC, non-HBOC) | | | | | Likely Pathogenic | | Likely Pathogenic | |
|  | c.1031delT | | p.Leu344TrpfsTer3 | | | Deletion, frameshift | | | **-** | | 1, HBOC | | | | | Pathogenic | | Likely Pathogenic | |
|  | c.1039A>G | | p.Met347Val | | | SNV, missense | | | rs769436449 | | 1, HBOC | | | | | VUS | | VUS | |
|  | c.1100delC | | p.Thr367MetfsTer15 | | | Deletion, frameshift | | | rs555607708 | | 1, HBOC | | | | | Pathogenic | | Pathogenic | |
|  | c.1684C>G | | p.Arg562Gly | | | SNV, missense | | | **-** | | 1, HBOC | | | | | Pathogenic, Likely Pathogenic | | VUS | |
|  | **Table S2. (Continued).** | | | | | |  | | |  | |  | | |  | | |  | |
|  |  | | |  | | |  | | |  | |  | | |  | | |  | |
| **Gene name** | **Sequence Variant Nomenclature on the cDNA level according to HGVS*** | | | **Sequence Variant Nomenclature on the amino acid level according to HGVS** | | | **Variant Type, Molecular Consequence** | | | **Variant rsID (if exist)** | | **Nr of Patients Carrying the Variant, Cohort** | | | **ClinVar Interpretation** | | | **Franklin Interpretation** | |
| *BRCA2* | c.658_659delGT | | | p.Val220fs | | | Deletion, frameshift | | | rs80359604 | | 1, HBOC | | | Pathogenic | | | Pathogenic | |
|  | c.1012G>A | | | p.Ala338Thr | | | SNV, missense | | | rs80358396 | | 1, non-HBOC | | | VUS | | | VUS | |
|  | c.1408dupG | | | p.Glu470fsTer6 | | | Duplication, frameshift | | | rs80359284 | | 2, HBOC | | | Pathogenic | | | Pathogenic | |
|  | c.1909+2T>C | | | - | | | SNV, Splice site variant | | | rs876658577 | | 1, HBOC | | | Pathogenic | | | Pathogenic | |
|  | c.3042T>G | | | p.Asn1014Lys | | | SNV, missense | | | rs1593898407 | | 1, HBOC | | | VUS | | | VUS | |
|  | c.5645C>A | | | p.Ser1882Ter | | | SNV, nonsense | | | rs80358785 | | 1, HBOC | | | Pathogenic | | | Pathogenic | |
|  | c.6613G>A | | | p.Val2205Met | | | SNV, missense | | | rs80358889 | | 1, HBOC | | | VUS | | | VUS | |
|  | c.6644_6647delACTC | | | p.Tyr2215fs | | | Deletion, frameshift | | | rs80359616 | | 1, HBOC | | | Pathogenic | | | Pathogenic | |
|  | c.6957delA | | | p.Arg2319SerfsTer9 | | | Deletion, frameshift | | | **-** | | 1, HBOC | | | Pathogenic | | | Likely Pathogenic | |
|  | c.7975A>G | | | p.Arg2659Gly | | | SNV, missense | | | rs80359026 | | 1, HBOC | | | Pathogenic | | | Pathogenic | |
|  | c.7976G>A | | | p.Arg2659Lys | | | SNV, missense | | | rs80359027 | | 1, HBOC | | | Pathogenic | | | Pathogenic | |
|  | c.8249_8251del | | | p.Lys2750del | | | Deletion, in-frame | | | rs80359703 | | 1, HBOC | | | Pathogenic | | | Likely Pathogenic | |
|  | c.8755-1G>A | | | - | | | SNV, Splice site variant | | | rs81002812 | | 1, 1 (HBOC, non-HBOC) | | | Pathogenic | | | Pathogenic | |
|  | c.9097dupA | | | p.Thr3033AsnfsTer11 | | | Duplication, frameshift | | | rs397507419 | | 1, HBOC | | | Pathogenic | | | Pathogenic | |
|  | c.9117G>A | | | p.Pro3039Pro | | | SNV, synonymous | | | rs28897756 | | 1, HBOC | | | Pathogenic | | | Likely Pathogenic | |
|  | c.9371A>T | | | p.Asn3124Ile | | | SNV, missense | | | rs28897759 | | 1, non-HBOC | | | Pathogenic | | | Likely Pathogenic | |
| *PALB2* | c.1250C>A | | | p.Ser417Tyr | | | SNV, missense | | | rs45510998 | | 1,1 (HBOC, non-HBOC) | | | VUS | | | VUS | |
|  | c.2167_2168delAT | | | p.Met723ValfsTer21 | | | Deletion, frameshift | | | rs587776416 | | 1, HBOC | | | Pathogenic | | | Pathogenic | |
|  | c.3217G>T | | | p.Val1073Phe | | | SNV, missense | | | rs1555458226 | | 1, HBOC | | | VUS | | | VUS | |
|  | | **Table S2. (Continued).** | | |  | | |  |  | | | |  |  | | |  | |  |
|  | |  | | |  | | |  |  | | | |  |  | | |  | |  |
| **Gene name** | | **Sequence Variant Nomenclature on the cDNA level according to HGVS*** | | | **Sequence Variant Nomenclature on the amino acid level according to HGVS** | | | **Variant Type, Molecular Consequence** | **Variant rsID (if exist)** | | | | **Nr of Patients Carrying the Variant, Cohort** | **ClinVar Interpretation** | | | **Franklin Interpretation** | |  |
| *ATM* | | c.1550T>C | | | p.Val517Ala | | | SNV, missense | rs777986229 | | | | 1, HBOC | VUS | | | VUS | |  |
|  |  | c.2250G>A | | | p.Lys750Lys | | | SNV, synonymous | rs1137887 | | | | 1, HBOC | Pathogenic, Likely Pathogenic | | | Pathogenic | |  |
|  |  | c.3154-2A>G | | | - | | | SNV, Splice site variant | rs730881357 | | | | 1, HBOC | Pathogenic | | | Pathogenic | |  |
|  |  | c.3802delG | | | p.Val1268Ter | | | SNV, nonsense | rs587779834 | | | | 1, HBOC | Pathogenic | | | Pathogenic | |  |
|  |  | c.5258A>G | | | p.Tyr1753Cys | | | SNV, missense | rs777481236 | | | | 1, HBOC | VUS | | | VUS | |  |
|  |  | c.5890A>G | | | p.Lys1964Glu | | | SNV, missense | rs201963507 | | | | 1, HBOC | VUS | | | VUS | |  |
|  |  | c.8228C>T | | | p.Thr2743Met | | | SNV, missense | rs730881321 | | | | 1, HBOC | VUS | | | VUS | |  |
| *RAD51C* | | c.323A>G | | | p.Asp108Gly | | | SNV, missense | rs1555593745 | | | | 1, HBOC | VUS | | | VUS | |  |
|  |  | c.502A>T | | | p.Arg168Ter | | | SNV, nonsense | rs587781490 | | | | 1, HBOC | Pathogenic | | | Pathogenic | |  |
|  |  | c.862A>G | | | p.Thr288Ala | | | SNV, missense | rs587781574 | | | | 1, non-HBOC | VUS | | | VUS | |  |
| *MSH6* | | c.2315G>A | | | p.Arg772Gln | | | SNV, missense | rs63750725 | | | | 1, non-HBOC | VUS | | | Likely Pathogenic | |  |
|  |  | c.3261dupC | | | p.Phe1088fsTer5 | | | Duplication, frameshift | rs267608078 | | | | 1, non-HBOC | Pathogenic | | | Pathogenic | |  |
|  |  | c.3961A>G | | | p.Arg1321Gly | | | SNV, missense | rs41295278 | | | | 1, HBOC | VUS | | | VUS | |  |

|  | **Table S2. (continued).** |  |  |  |  |  |  |
| --- | --- | --- | --- | --- | --- | --- | --- |
|  |  |  |  |  |  |  |  |
| **Gene name** | **Sequence Variant Nomenclature on the cDNA level according to HGVS*** | **Sequence Variant Nomenclature on the amino acid level according to HGVS** | **Variant Type, Molecular Consequence** | **Variant rsID (if exist)** | **Nr of Patients Carrying the Variant, Cohort** | **ClinVar Interpretation** | **Franklin Interpretation** |
| *APC* | c.5689G>T | p.Ala1879Ser | SNV, missense | rs587779799 | 1, HBOC | VUS | VUS |
| *BARD1* | c.1339C>G | p.Leu447Val | SNV, missense | rs376727038 | 1, HBOC | VUS | VUS |
|  | c.1690C>T | p.Gln564Ter | SNV, nonsense | rs587780021 | 1, HBOC | VUS | Pathogenic |
| *BLM* | c.1642C>T | p.Gln548Ter | SNV, nonsense | rs200389141 | 1, HBOC | Pathogenic | Pathogenic |
| *CDH1* | c.2499delT | p.Phe833fsTer13 | Deletion, frameshift | **-** | 1, HBOC | not known | Pathogenic |
| *ERCC2* | c.2041G>A | p.Asp681Asn | SNV, missense | rs121913023 | 1, HBOC | Pathogenic | Likely pathogenic |
| *FANCI* | c.706T>C | p.Phe236Leu | SNV, missense | **-** | 1, HBOC | VUS | VUS |
|  | c.1412C>G | p.Pro471Arg | SNV, missense | **-** | 1, HBOC | VUS | VUS |
| *PMS2* | c.2206G>A | p.Glu736Lys | SNV, missense | **-** | 1, HBOC | VUS | VUS |
| *KIT* | c.757-9A>G | - | SNV, Splice site variant | rs367986084 | 1, HBOC | VUS | VUS |
| *RET* | c.2348A>C | p.Asn783Thr | SNV, missense | rs587778656 | 1, HBOC | VUS | VUS |
| *MUTYH* | c.1187G>A | p.Gly396Asp | SNV, missense | - | 1, non-HBOC | Pathogenic/Likely Pathogenic | Likely Pathogenic |
| *EPCAM* | c.577G>A | p.Ala193Thr | SNV, missense | - | 1, non-HBOC | VUS | VUS |
| *FANCD2* | c.2803_2804delAT | p.Ile935SerfsTer14 | Deletion, frameshift | rs751895071 | 1, non-HBOC | VUS | VUS |
| *POLE* | c.325C>T | p.Arg109Ter | SNV, nonsense | **-** | 1, non-HBOC | Pathogenic, Likely Pathogenic | Pathogenic |
| *ERCC3* | c.325C>T | p.Arg109Ter | SNV, nonsense | rs34295337 | 1, non-HBOC | Pathogenic, Likely Pathogenic | Pathogenic |
| *PTCH1* | c.901G>A | p.Asp300Asn | SNV, missense | rs767601899 | 1, non-HBOC | VUS | VUS |
| *RAD50* | c.287T>C | p.Val96Ala | SNV, missense | rs1554097576 | 1, HBOC | VUS | VUS |
| *RAD51B* | c.2T>C | p.Met1Thr | SNV, missense | rs142567687 | 1, HBOC | VUS | VUS |
| HGVS: Human Genome Variation Society; VUS: variant of uncertain significance; HBOC cohort: hereditary breast and ovarian cancer; non-HBOC cohort: healthy persons with hereditary cancer relatives | | | | | | | |
